# Annular Erythematous Patches as the Presenting Sign of Extranodal Natural Killer/T-Cell Lymphoma

**DOI:** 10.4274/tjh.2016.0071

**Published:** 2016-12-01

**Authors:** Can Baykal, Algün Polat Ekinci, Şule Öztürk Sarı, Zeynep Topkarcı, Özgür Demir, Nesimi Büyükbabani

**Affiliations:** 1 İstanbul University İstanbul Faculty of Medicine, Department of Dermatology and Venereology, İstanbul, Turkey; 2 İstanbul University İstanbul Faculty of Medicine, Department of Pathology, İstanbul, Turkey; 3 Bakırköy Dr. Sadi Konuk Training and Research Hospital, Clinic of Dermatology, İstanbul, Turkey

**Keywords:** Extranodal natural killer/T cell lymphoma, Erythematous indurated plaques, Annular erythematous patch, Annular erythema

## To the Editor,

Extranodal natural killer/T-cell lymphoma (ENKTL) is a distinct type of lymphoma strongly associated with Epstein-Barr virus (EBV) infection and showing an aggressive course [1]. It usually presents as a localized disease in the upper aerodigestive tract, from the nasal cavity to the hypopharynx [2,3], but it may rapidly extend to the neighboring tissues and disseminate to various organs such as the small intestine, epiglottis, testes, adrenal gland, kidneys, and breasts [4,5]. As nasal/upper aerodigestive tract involvement may only cause nonspecific symptoms in the early period, diagnosis may be initially established based upon skin lesions [6]. We present two ENKTL patients with unusual dermatological findings.

**Figure 1:**
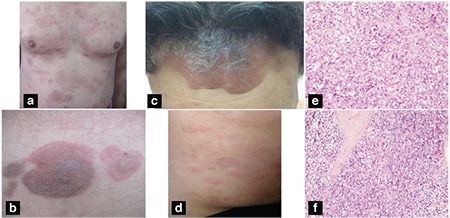
a, b) Widespread eruption on the trunk consisting of annular erythematous patches (Patient 1). c) Infiltrated plaque on the forehead extending to the scalp (Patient 2). d) Annular erythematous patches and purpuric patches circumscribed with a thin erythematous ring (Patient 2). e) Dense neoplastic infiltration of atypical lymphocytes on the mid-deep dermis (hematoxylin and eosin, 200x). f) In situ hybridization for EBER shows positive signals (EBER, 100x) (Patient 2).

Patient 1, a 44-year-old male, presented with a widespread eruption on the trunk, scalp, and arms consisting of annular erythematous patches (Figure 1a) and hyperpigmented/purpuric patches circumscribed with erythematous rings (Figure 1b). A biopsy revealed neoplastic infiltration of atypical lymphocytes expressing CD56 and granzyme-B but negative for CD2, CD3, CD8, and CD20. Nasopharyngeal involvement was suspected with radiologic imaging (magnetic resonance imaging) and ENKTL was diagnosed after a nasopharyngeal biopsy. Bone marrow biopsy was normal. Following CHOP chemotherapy, most of the cutaneous lesions resolved with slight hyperpigmentation, but complete clearance was not achieved during the 3-month follow-up period.

Patient 2, a 39-year-old male having a history of infectious mononucleosis 5 months earlier, presented with widespread infiltrated plaques on the nose, cheeks, (Figure 1c), forehead, scalp, trunk, and arms and a deep nodule on the hard palate for 2 months. Annular erythema and purpuric patches circumscribed with annular rims were remarkable on the back (Figure 1d). Serum EBV-PCR and EBV VCA-IgG tests revealed positive results. Punch biopsies performed from both erythematous patches on the back and infiltrated plaques showed neoplastic lymphocytic infiltration with EBV-encoded RNA (EBER) positivity by in situ hybridization, which confirmed the diagnosis of ENKTL (Figures 1e and 1f). A PET-CT examination revealed nasopharynx, palate, and tonsil involvements and metastatic parenchymatous nodules in both lungs.

A broad spectrum of skin lesions such as erythematous indurated plaques, painful subcutaneous nodules, persistent cellulitis-like or abscess-like swellings, panniculitis-like lesions, mycosis fungoides-like lesions, and nonhealing ulcers can be seen in patients with ENKTL [7,8,9]. Three ENKTL cases were reported in which patients presented with skin lesions on the trunk and extremities described as infiltrated erythema, edematous erythema, and dark red erythema, one of them showing an annular configuration [8]. An ENKTL case also involving erythematous patches that developed and regressed over the course of chemotherapy was reported [10]. However, this was considered as a possible paraneoplastic sign.

Both of our patients had unusual lesions for cutaneous lymphoma, namely erythematous patches mostly showing annular configurations besides the more typical infiltrated plaques of Patient 2. From a clinical standpoint, the appearance of these erythematous lesions is like an inflammatory disease and may be a paraneoplastic sign. However, the lesions were nonmigratory and had persisted for a long time, in contrast to the expected course of possible reactive inflammatory dermatoses. Moreover, in both cases histopathologic examination showed neoplastic infiltration of ENKTL.

In conclusion, persistent erythematous patches with annular shape may be among the skin involvement patterns of ENKTL and awareness of this peculiar finding may avoid delay in its diagnosis.
